# Dissection of differential vanadate sensitivity in two *Ogataea* species links protein glycosylation and phosphate transport regulation

**DOI:** 10.1038/s41598-018-34888-5

**Published:** 2018-11-06

**Authors:** Azamat V. Karginov, Anastasia V. Fokina, Hyun Ah Kang, Tatyana S. Kalebina, Tatyana A. Sabirzyanova, Michael D. Ter-Avanesyan, Michael O. Agaphonov

**Affiliations:** 1 Bach Institute of Biochemistry, Research Center of Biotechnology RAS, Moscow, Russian Federation; 2 Department of Life Science, Chung-Ang University, Seoul, Republic of Korea; 3 Department of Molecular Biology, Biological Faculty, Lomonosov Moscow State University, Moscow, Russian Federation

## Abstract

The closely related yeasts *Ogataea polymorpha* and *O*. *parapolymorpha* differ drastically from each other by sensitivity to the toxic phosphate analog vanadate. Search for genes underlying this difference revealed two genes, one designated as *ABV1* (*A*lcian *B*lue staining, *V*anadate resistance), which encodes a homologue of *Saccharomyces cerevisiae* Mnn4 responsible for attachment of mannosylphosphate to glycoside chains of secretory proteins, and the other designated as its *S*. *cerevisiae* homologue *PHO87*, encoding the plasma membrane low affinity phosphate sensor/transporter. The effect of Pho87 on vanadate resistance was bidirectional, since it decreased the resistance on phosphate-depleted medium, but was required for pronounced protection against vanadate by external phosphate. This highlights the dual function of this protein as a low affinity phosphate transporter and an external phosphate sensor. Involvement of Pho87 in phosphate sensing was confirmed by its effects on regulation of the promoter of the *PHO84* gene, encoding a high affinity phosphate transporter. The effect of Abv1 was also complex, since it influenced Pho87 level and enhanced repression of the *PHO84* promoter *via* a Pho87-independent pathway. Role of the identified genes in the difference in vanadate resistance between *O*. *polymorpha* and *O*. *parapolymorpha* is discussed.

## Introduction

Inorganic phosphate (P_i_) is a crucial nutrient for living cells. As the plasma membrane is impermeable to this cation, its uptake depends on special transporters. *Saccharomyces cerevisiae* possesses five P_i_ transporters: four plasma membrane transporters Pho84, Pho87, Pho89, and Pho90, which provide P_i_ uptake from environment in different conditions, and one vacuolar transporter Pho91, which is probably required for supplying the cytosol with phosphate from the vacuolar pool (for the review see^1,2^). The high affinity P_i_ transporters Pho84 and Pho89 differ from each other by pH optimum and cation requirements. Particularly, transport through Pho84 depends on protons and reaches the highest rate at pH 4.5, while Pho89 is a P_i_:Na^+^-coupled symporter with an optimum at pH 9.5^3^. The *PHO84* and *PHO89* genes are repressed at high external P_i_ concentration and activated upon P_i_ limitation^3,4^. Pho87, Pho90, and Pho91 are low affinity P_i_ transporters^5^. Unlike genes for the high affinity transporters, the *PHO87*, *PHO90*, and *PHO91* genes do not undergo tight regulation depending on P_i_ availability^6^. However, at low external P_i_ concentration, the presence of the plasma membrane low affinity P_i_ transporters can be detrimental to the cell, and this effect can be alleviated by targeting these proteins for vacuolar degradation *via* the endocytic pathway^7^.

The transcriptional response to changes in cytosolic P_i_ concentration is mediated by a complex regulatory circuit referred to as PHO pathway (for a review see^8^), which controls expression of certain genes through the activity of transcription factors Pho4 and Pho2^9^. Beside transcription factors, the core of this circuit involves a complex consisting of cyclin-dependent protein kinase (CDK) Pho85, one of its cyclins Pho80, and the CDK inhibitor Pho81. Upon P_i_ limitation conditions, Pho4 is relocalized to the nucleus where it is able to bind specific sites in promoter regions in cooperation with Pho2. Elevation of intracellular phosphate level relieves inhibition of Pho85 by Pho81^10,11^, leading to phosphorylation of Pho4 at multiple sites. This prevents Pho4 interaction with Pho2 and causes its redistribution from the nucleus to the cytosol^12–15^. Apart from this circuit, regulation of *PHO84*, which depends on the low affinity P_i_ transporters and does not depend on internal P_i_ concentration, has also been elucidated^16^. The high affinity transporter Pho84 and low affinity transporter Pho87 were shown to be P_i_ sensors allowing cells to respond to changes in external P_i_ availability *via* the protein kinase A dependent signaling pathway^17^.

Vanadate (orthovanadate, VO_4_^3−^) is a toxic P_i_ analog, which has multiple targets within the cell. It efficiently inhibits many different enzymes, whose substrates carry a phosphate moiety^18^. One can assume that multiple targets can be protected from this inhibitor only by prevention of its uptake or by rapid conversion into less toxic compounds. Indeed, vanadate resistant mutants isolated in *Candida albicans*^19^ and *Neurospora crassa*^20,21^ were defective in the P_i_ transport system. Interestingly, in the conventional yeast *S*. *cerevisiae*, an increase in vanadate resistance can be achieved by alterations in the Golgi apparatus protein glycosylation^22^. At the same time no mutations in the P_i_ transport system conferring this phenotype have been reported in this yeast.

Some yeast species can exhibit much higher resistance to vanadate than others. For example, compared to *S*. *cerevisiae*, *Ogataea* (*Hansenula*) *polymorpha* is much more resistant to this compound^23^, while its closest relative *O*. *parapolymorpha*^24^ is not^25^. Here we used these two yeast species to gain insight into the mechanisms involved in vanadate resistance and its relation to P_i_ transport and protein glycosylation.

## Results

### Inactivation of *YPT6* or a homologue of *S*. *cerevisiae MNN4* decrease vanadate resistance in *O*. *polymorpha* but not in *O*. *parapolymorpha*

To find genes responsible for high vanadate resistance in *O*. *polymorpha*, vanadate sensitive clones were selected among transformants obtained using linearized plasmids supposed to randomly integrate within the genome. Clones with pronounced vanadate sensitivity were subjected to identification of the plasmid integration loci. In two cases, involvement of the identified genes in vanadate resistance was confirmed by their targeted inactivation. One of them (accession number MH286258) codes for a homolog of the *S*. *cerevisiae* Ypt6 GTPase involved in retrograde vesicle transport between different compartments of the secretory pathway and endosomes^26^. Inactivation of this gene in *O*. *polymorpha* led to increased sensitivities to SDS and caffeine as well as to decrease in the carboxypeptidase Y level (Supplementary Fig. S1). In *O*. *parapolymorpha* this mutation caused hypersensitivities to caffeine, high salt concentration and elevated temperature (Supplementary Fig. S1). All these phenotypes have been previously observed in *S*. *cerevisiae ypt6* mutants^26,27^. Based on these observations, this gene was also designated as *YPT6*.

**Figure 1:**
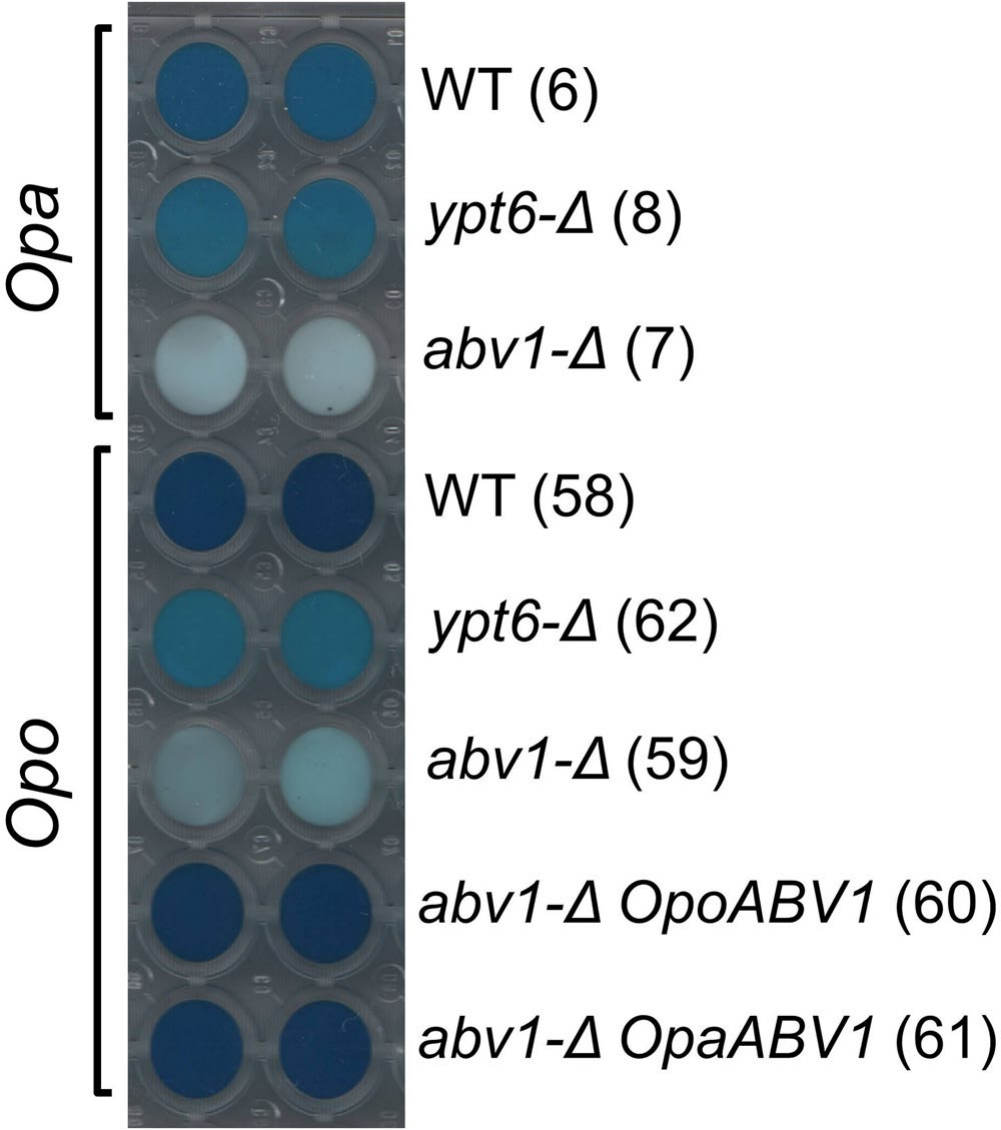
Staining of yeast cells with the Alcian blue dye. “*Opa”* and *“Opo”*, *O*. *parapolymorpha* and *O*. *polymorpha*, respectively; “WT”, transformants possessing *ABV1* and *YPT6* wild-type alleles; “*ypt6-Δ*” and “*abv1-Δ*”, transformants disrupted for *YPT6* or *ABV1*, respectively. “*OpoABV1*” and “*OpaABV1*” indicate presence of plasmids with the corresponding genes. Numbers in brackets correspond to designation of transformants in the Supplementary Table S1.

Another identified gene (accession number MH286257) encodes a homolog of *S*. *cerevisiae* Mnn4, which was shown to be involved in the attachment of mannosylphosphate to protein glycoside chains in the secretory pathway^28^. *O*. *polymorpha* cells were efficiently stained with the Alcian blue dye (Fig. 1), indicating presence of mannosylphosphate in the glycoside chains of the cell wall^29^, while inactivation of the identified gene led to inability to bind Alcian blue indicating drastic decrease or absence of mannosylphosphate in the cell wall. Designation ‘*MNN4*’ for the identified gene seemed to be objectionable, since *O*. *polymorpha*^30^ and *O*. *parapolymorpha*^31^ genomes possess another ORF, which codes for a protein (accession number XP_013933535.1) showing even higher similarity to *S*. *cerevisiae* Mnn4 since its sequence alignment reveals 195 identical positions of 780 amino acid sequence compared to 150 of 783 residues in the identified gene. To avoid this ambiguity, the gene was designated *ABV1* (Alcian Blue staining, Vanadate resistance), according to the observed mutant phenotypes.

Inactivation of *YPT6* also led to some decrease in Alcian blue binding (Fig. 1), which might lead one to suggest that its effect on the vanadate sensitivity might be related to alteration of phosphomannosylation of glycoside chains in the secretory pathway. However, this was not supported by further experiments.

**Figure 2:**
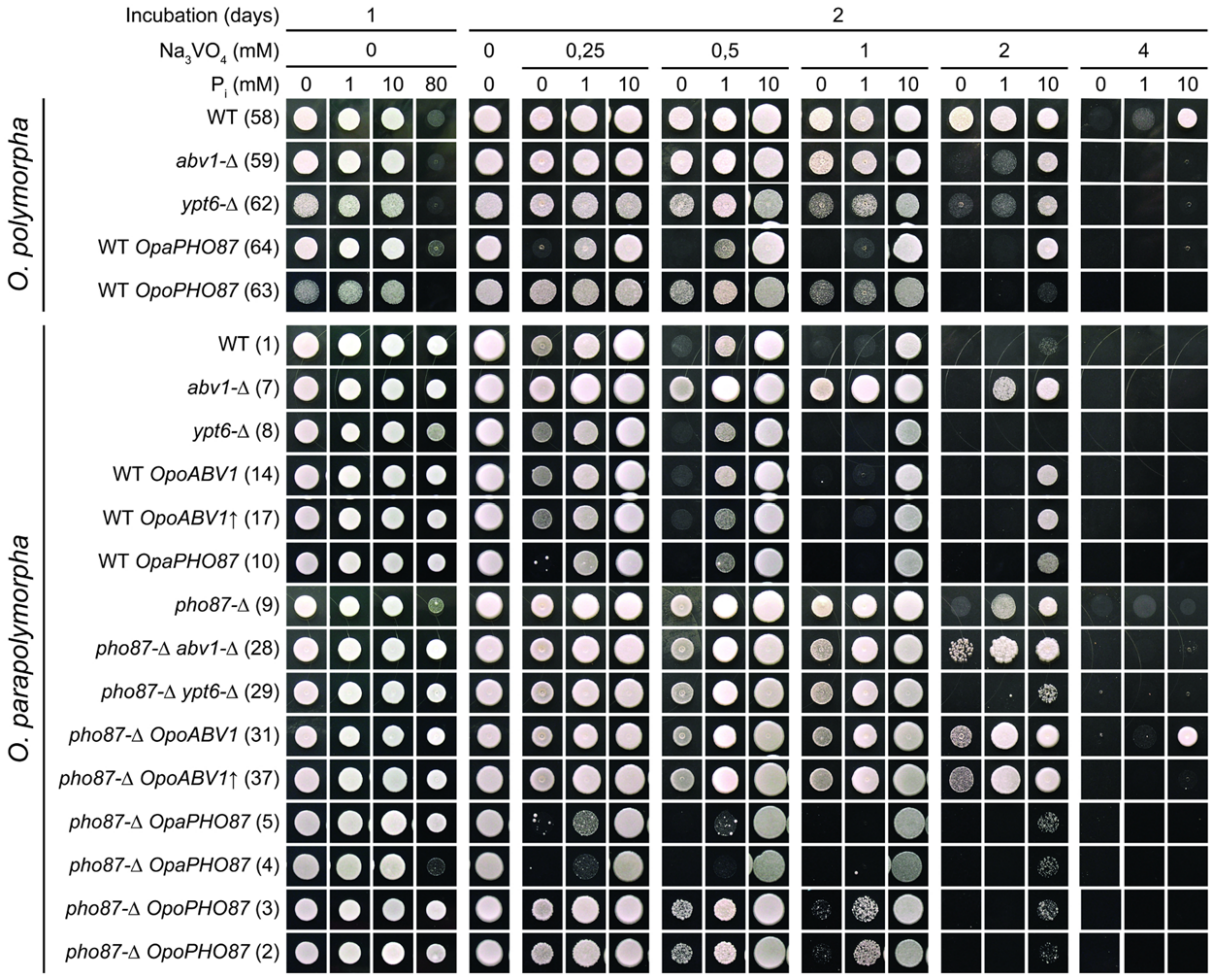
Dependence of vanadate sensitivity on phosphate concentration in culture medium. Numbers in brackets correspond to designation of transformants in the Supplementary Table S1. Overnight saturated cultures grown in the phosphate depleted medium supplemented with 0.5 mM Na-Pi buffer were 500-fold diluted and spotted onto plates with the phosphate depleted medium supplemented with different concentrations of Na_3_VO_4_ and Na-P_i_ buffer pH 7.2.

Inactivation of *YPT6* in the sensitive species *O*. *parapolymorpha* did not noticeably affected vanadate resistance, while effect of *ABV1* inactivation was opposite to that in *O*. *polymorpha*, since this mutation increased vanadate resistance of the former species (Fig. 2, *O*. *polymorpha* and *O*. *parapolymorpha* strains labeled “WT”, “*abv1-Δ*” and “*ypt6-Δ*”).

### *O*. *parapolymorpha* vanadate resistance depends on *ABV1* gene dosage

As *O*. *polymorpha* and *O*. *parapolymorpha* genomes are almost identical, one could suggest that functions of only one or several genes may determine their difference in vanadate resistance. To search for *O*. *polymorpha* genes, which are able to improve vanadate resistance in *O*. *parapolymorpha*, the *O*. *parapolymorpha* DL1-L strain was transformed with an *O*. *polymorpha* genomic library. Several vanadate resistant transformants were selected. All of them possessed plasmids containing the *ABV1* gene. This led us to conclude that function of *ABV1* is insufficient in this yeast. However, the untransformed *O*. *parapolymorpha* cells were able to bind Alcian blue, while inactivation of *ABV1* abolished the staining. At the same time, the staining of *O*. *parapolymorpha* wild-type cells seemed not to be as intensive as in *O*. *polymorpha* (Fig. 1).

The *ABV1* gene products of these two yeast species are not completely identical (7% of mismatches), which might lead one to suggest that *O*. *parapolymorpha* Abv1 is functional in the phosphomannosylation of cell wall proteins, but does not target a specific subset of proteins including ones involved in increasing vanadate resistance. However, the autonomously replicating plasmid with *O*. *parapolymorpha ABV1* was able to increase vanadate resistance like its *O*. *polymorpha* orthologue in both, *O*. *polymorpha abv1-Δ* and *O*. *parapolymorpha* (Supplementary Fig. S2). This indicates that ability to increase vanadate resistance is not due to some special property of *O*. *polymorpha* Abv1, but rather depends on its production level.

Introduction of a multi copy plasmid with *ABV1* into *O*. *parapolymorpha* cells led to only a moderate increase in vanadate resistance, which was still far from that of the vanadate resistant species *O*. *polymorpha*, indicating that this gene is not the only player that determines this difference between the two yeast species. According to our previous experience, the genomic library we used in this approach is representative enough to cover the whole *O*. *polymorpha* genome. Indeed, using it, we have always been able to clone target genes, some of which were toxic in *Escherichia coli*, possibly restricting their representation in the library. This led us to suggest that lower vanadate resistance in *O*. *parapolymorpha* compared to *O*. *polymorpha* is prompted by increased activity of some proteins(s) and the current approach does not allow identification of additional player(s) determining the difference in vanadate resistance between these yeasts.

### Plasma membrane low affinity phosphate transporter/sensor Pho87 modulates vanadate resistance in *O*. *parapolymorpha*

One could expect that inactivation of genes, whose increased expression is responsible for the lower vanadate resistance of *O*. *parapolymorpha*, should improve the resistance. To find such genes we mutagenized *O*. *parapolymorpha* by random integration of a linearized plasmid (see Materials and Methods) and selected vanadate resistant clones. Plasmid integration in one of the selected clones, which showed pronounced and stable vanadate resistance, occurred in the gene coding for a homologue of the *S*. *cerevisiae* plasma membrane low affinity P_i_ transporters Pho87 and Pho90 (accession number XP_013932315). In contrast to *S*. *cerevisiae*, *O*. *parapolymorpha*^31^ and *O*. *polymorpha*^30^ genomes possess only one gene coding for such transporter, since only two ORFs with sufficient similarity to *S*. *cerevisiae PHO87* can be revealed: one of which is the identified gene, while another one (accession number XP_013933495) codes for the homologue of the vacuolar P_i_ transporter Pho91. This and additional data described below allowed us to designate the identified gene like its *S*. *cerevisiae* homologue *PHO87*. Interestingly, this gene is likely to be essential in *O*. *polymorpha*, since we were unable to inactivate *PHO87* in a strain carrying only one allele of this gene, while we could do this in transformants with an integrative plasmid bearing *O*. *parapolymorpha PHO87* (*OpaPHO87*).

**Figure 3:**
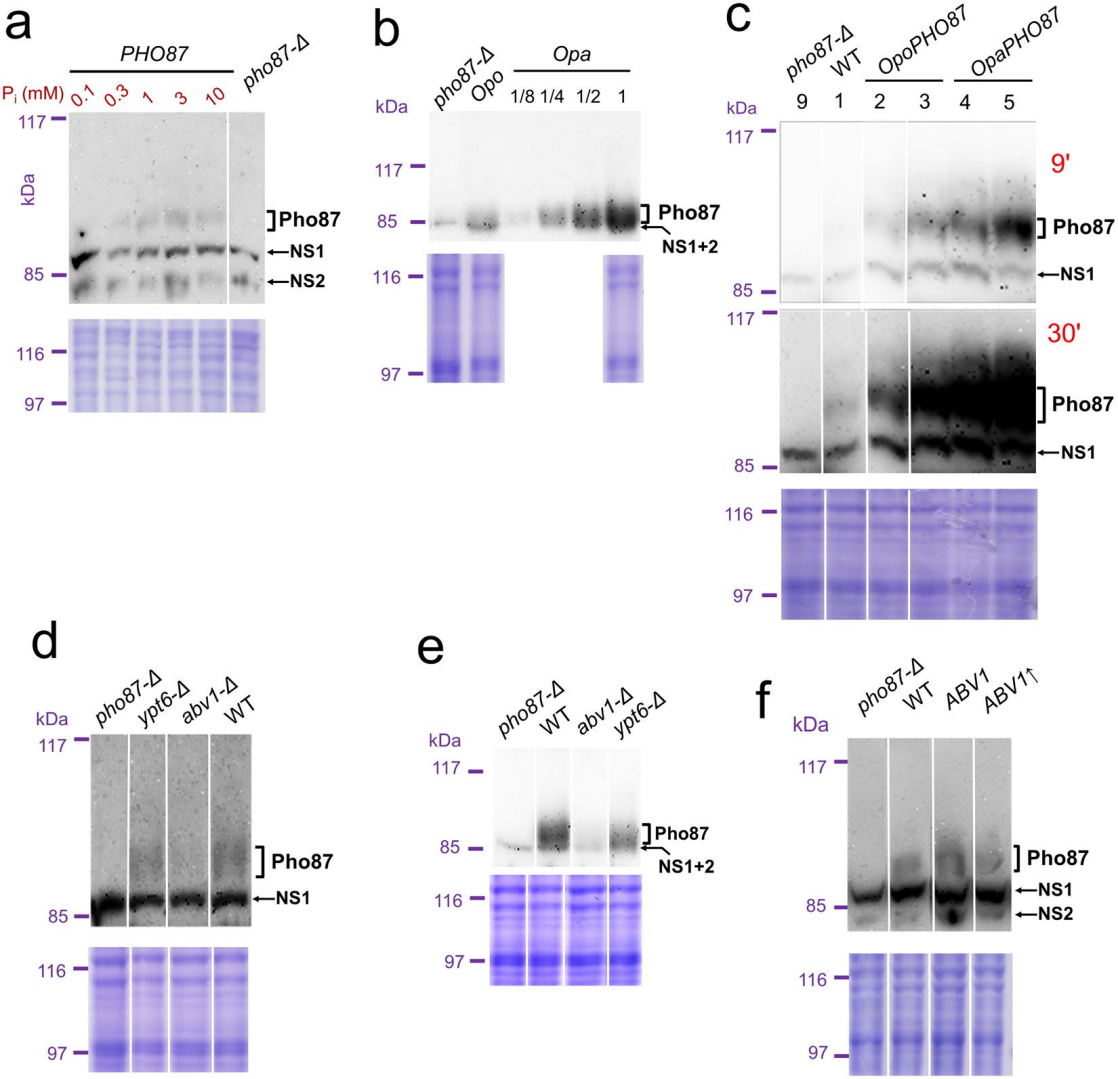
Immunoblotting of Pho87 (upper panels) and Coomassie stained gels as loading controls (lower panels). Lanes cropped from different parts of the same gel or blot are divided by spaces. Positions of the marker bands are shown at the left. In Coomassie-stained gels the NEB marker (Cat. #P7702) was used; the prestained marker (ThermoFisher Scientific, cat. #sm0441) was used in immunoblots. Despite the affinity purification, some non-specific bands (NS1 and NS2) migrating close to the full-length Pho87 band were still revealed by this antibody. To resolve them, in case of blots presented in panels a, c, d, and f electrophoresis was run until the 85 kDa pre-stained marker band came to end of the gel. *O*. *parapolymorpha pho87-Δ* strain (#9 in the Supplementary Table S1) was used in all cases. (**a**) Analysis of Pho87 content in *O*. *parapolymorpha* grown in medium repleted with specified phosphate concentrations. “*PHO87*”, stain #6 (Supplementary Table S1); “*pho87-Δ* ”, the negative control strain, which was grown in the medium repleted with 1 mM P_i_. (**b)** Comparison of Pho87 content in *O*. *polymorpha* and *O*. *parapolymorpha* cells grown in regular YPD. “Opo”, “Opa” and “*pho87-Δ*” correspond to strains ## 58, 6, 9 in the Supplementary Table S1, respectively. “1/8”, “1/4”, “1/2” and “1” indicate that sample was 8-, 4-, 2-fold diluted, or undiluted, respectively. (**c**) Expression of the plasmid-borne *OpaPHO87* and *OpoPHO87* in *O*. *polymorpha pho87-Δ* strain. Lanes are marked with the strain numbers according to the Supplementary Table S1. Two expositions (9′ and 30′) of the immunoblot are presented. (**d**) Effects of *ABV1* and *YPT6* deletions in *O*. *polymorpha*. WT, *abv1-Δ*, *ypt6-Δ*, and *pho87-Δ*, strains designated as #58, 59, 62, and 9 in the Supplementary Table S1, respectively. (**e**) Effects of *ABV1* and *YPT6* deletions in *O*. *parapolymorpha*. *pho87-Δ*, WT, *abv1-Δ*, and *ypt6-Δ*, strains designated as #9, 6, 7, and 8 in the Supplementary Table S1, respectively. (**f**) Effect of *ABV1* extra copies in *O*. *parapolymorpha*. *pho87-Δ*, WT, *ABV1*, and *ABV1*↑, strains designated as #9, 6, 14, and 17 in the Supplementary Table S1, respectively.

As was shown in *S*. *cerevisiae*, low affinity P_i_ transporters support phosphate uptake at a relatively high external phosphate concentration, while their abundance at low external P_i_ concentrations can be deleterious^32^. This is also probably true in *O*. *parapolymorpha* since a drastic drop in Pho87 amount was observed when cells were incubated in phosphate depleted medium (Fig. 3a). This was not accompanied by substantial repression of the *OpaPHO87* promoter (Table 1) indicating that the amount of this protein might be regulated by vacuolar degradation, like in *S*. *cerevisiae*^7^.

**Table 1:** Relative activities of β-galactosidase expressed under control of the *PHO87* promoter in cells grown in phosphate depleted medium with (High P_i_) or without (Low P_i_) supplementation with 10 mM Na-P_i_ buffer. Several strain numbers are indicated if mean value was calculated using independently obtained clones. If only one clone was used, the mean value was calculated from activities in 3 independently obtained cultures.

Essential genotype and number in the Table S1	Low P_i_	High P_i_
WT (45)	100 ± 8	106 ± 13
*abv1* (46)	75 ± 5	40 ± 7
*OpoABV1* (48–50)	105 ± 18	124 ± 19
*OpoABV1*↑ (51–54)	67 ± 5	44 ± 16
*PHO87*↑ (55–57)	109 ± 24	88 ± 18
*pho87* (47)	211 ± 39	79 ± 12

Importantly, vanadate resistance depended on the *PHO87* gene dosage since introducing a plasmid with this gene made *O*. *polymorpha* and *O*. *parapolymorpha* cells more sensitive to vanadate (Fig. 2), at least at low P_i_ concentration. One could assume that the difference in vanadate resistance between these yeasts is determined by a difference in *PHO87* expression levels. In agreement with this, amount of Pho87 in the resistant species *O*. *polymorpha* was much lower than in *O*. *parapolymorpha* if cells were incubated in regular YPD medium (Fig. 3b). It was also possible that *Opo*Pho87 is less efficient as a transporter than *Opa*Pho87. Indeed, introduced into the *O*. *parapolymorpha pho87-Δ* strain, the resistant species gene *OpoPHO87* conferred less sensitivity than its native gene, *OpaPHO87*, not only in clones with approximately the same expression levels (Figs 2 and 3c; transformant 3 *vs*. transformant 4), but even when *OpoPHO87* was more highly expressed (transformants 2 and 3 *vs*. transformant 1 having the wild-type *OpaPHO87* locus). Interestingly, *OpaPHO87* expression level in transformant 4 was noticeably lower than in 5, but their vanadate sensitivities were similar, indicating that when Pho87 content reaches a certain level its alteration is unable to noticeably affect vanadate sensitivity.

Inactivation of *PHO87* in *O*. *parapolymorpha* did not increase vanadate resistance to the same level as in *O*. *polymorpha* wild-type cells (Fig. 2) suggesting the presence of an additional mechanism underlying this difference. One can suggest that vanadate, being a phosphate analog, enters the cell *via* phosphate transporters. In this case, vanadate toxicity should depend on the phosphate concentration in culture medium. In agreement with this suggestion, increasing phosphate concentration rescued cells from vanadate toxicity, though this effect became much less pronounced after inactivation of *PHO87* (Fig. 2). Transformants overexpressing *OpaPHO87* or *OpoPHO87* showed reduced vanadate resistance on phosphate depleted medium and efficient protection against vanadate by increased phosphate concentration. This dual effect of Pho87 on the sensitivity could originate from its two functions, namely a low affinity phosphate transporter and external phosphate sensor. In terms of this hypothesis, Pho87 confers vanadate uptake as a phosphate transporter and inhibits it as an external phosphate sensor by repressing another plasma membrane transporter, which is also responsible for vanadate uptake.

Based on the homology with *S*. *cerevisiae* high affinity plasma membrane phosphate transporters, *O*. *polymorpha* and *O*. *parapolymorpha* genomes also possess genes for these transporters – one for Pho84 and one for Pho89 (accession numbers XP_013935929 and XP_013933062, respectively). Importantly, as it was shown in *S*. *cerevisiae*, Pho87 and Pho84 have higher activity at lower pH, while Pho89 is active at higher pH and requires Na^+^ cations^33^. Analyzing ^32^P_i_ uptake in *O*. *parapolymorpha* we observed that at pH4.5 its rate was higher (approx. 5.0 fold in strain possessing wild-type *PHO87* locus and approx. 2 fold in the *Δpho87* strain) than at pH8.0 and in presence of 10 mM NaCl (Supplementary Table S2). This indicates that like in *S*. *cerevisiae*^17^, *O*. *parapolymorpha* Pho84 has more impact on phosphate uptake than Pho89.

To study *PHO84* transcription regulation we fused its promoter to the ORF encoding *Renilla* luciferase. Indeed, *PHO84* promoter provided significantly different gene expression levels at high and low phosphate concentration in culture medium. This regulation apparently depended on *PHO87*, since its deletion affected the promoter repression and activation at high and low phosphate concentration, respectively, while its overexpression decreased *PHO84* promoter activity in both cases (Table 2). In addition, production of acid phosphatase, which is known to depend on internal phosphate concentration^34^, was studied (Table 2). In contrast to the luciferase produced under control of the *PHO84* promoter, regulation of production of acid phosphatase was almost unaffected by inactivation or extra copies of *PHO87*. In particular, acid phosphatase production was efficiently repressed in the wild type strain at high phosphate concentration, while both inactivation and extra copies of *PHO87* led to very slight derepression.

**Table 2:** Relative activities of *Renilla* luciferase expressed under control of *PHO84* promoter (*P*_*PHO84*_) and of acid phosphatase (AP) in cells grown in the phosphate depleted medium with (High P_i_) or without (Low P_i_) supplementation with 10 mM Na-P_i_ buffer. Several strain numbers are indicated if mean value was calculated using independently obtained clones. If only one clone was used, the mean value was calculated from activities in 4 independently obtained cultures.

Essential genotype and number in the Supplementary Table S1	Low P_i_		High P_i_	
	*P*_*PHO84*_	AP	*P*_*PHO84*_	AP
WT (6)	100 ± 6	100 ± 2	2.03 ± 0.04	<0.03
*abv1* (7)	317 ± 16	112 ± 5	0.61 ± 0.23	<0.03
*OpoABV1* (14–16)	81 ± 18	114 ± 5	1.49 ± 0.62	<0.03
*OpoABV1*↑ (17–20)	66 ± 5	118 ± 4	1.33 ± 0.49	0.18 ± 0.02
*PHO87* (10–13)	2.31 ± 0.03	137 ± 2	1.08 ± 0.05	0.33 ± 0.06
*pho87* (9)	3.9 ± 0.4	94 ± 3	13.3 ± 3.0	0.041 ± 0.01
*pho87 abv1* (28)	246 ± 25	85 ± 4	6.5 ± 1.4	<0.03
*pho87 OpoABV1* (31–36)	0.04 ± 0.015	105 ± 11	0.046 ± 0.019	0.12 ± 0.022
*pho87 OpoABV1↑* (37–40)	57 ± 11	108 ± 5	6.8 ± 1.1	<0.03
*ypt6* (8)	83 ± 7	n.d.	2.2 ± 0.2	n.d.
*ypt6 abv1* (27)	286 ± 44	n.d.	0.6 ± 0.06	n.d.
*ypt6 pho87* (29)	1.9 ± 0.2	n.d.	4.4 ± 0.5	n.d.
*ypt6 pho87 abv1* (30)	277 ± 25	n.d.	6.5 ± 1.8	n.d.

### *ABV1* affects regulation of phosphate homeostasis

Unlike Pho87, which could be directly involved in vanadate uptake as a phosphate transporter and sensor, the role of mannosylphosphate attachment to glycoproteins catalyzed by Abv1 in modulation of vanadate resistance was not obvious. It was possible to suggest that this modification somehow influences regulation of phosphate homeostasis. To test this hypothesis, we studied effects of both *ABV1* deletion and dosage increase on vanadate sensitivities (Fig. 2) and regulation of *PHO84*, and *PHO5* genes (Table 2) in relation to phosphate concentration in culture medium.

Importantly, transformants bearing extra copies of *ABV1* were obtained by two different approaches: (i) targeting of single copy plasmid integration into the *LEU2* locus and (ii) using a special autonomously replicating vector allowing selection of subclones that arose due to integration of several plasmid copies into the genome (see Materials and Methods).

In the *O*. *parapolymorpha* strain with the *PHO87* wild-type allele, multiple (but not single copy) integration of an *OpoABV1* containing plasmid led to some derepression of the *PHO5* gene at high phosphate concentration in culture medium indicating reduction of cytosolic phosphate content (Table 2). Despite this difference, the effects of multiple and single copy integration on vanadate resistance were undistinguishable. At the same time, in a strain with disrupted *PHO87*, one extra copy of *ABV1* led to some *PHO5* derepression at high phosphate concentration and had more impact on vanadate resistance than the multi copy plasmid. This correlated with stronger repression of the *PHO84* promoter. These results demonstrate that *ABV1* is able to affect response to external phosphate and to modulate phosphate uptake in absence of *PHO87*.

Effect of the *ypt6-Δ* mutation on the *PHO84* promoter regulation was opposite to the effect of *abv1-Δ* (Table 2), despite similar effects on vanadate resistance. This indicates that *ypt6-Δ* may affect vanadate resistance independently of *ABV1*.

In contrast to observations in the resistant species *O*. *polymorpha*, inactivation of *ABV1* in the sensitive species *O*. *parapolymorpha* led to a noticeable increase in the vanadate resistance, which even exceeded the resistance increase in response to *ABV1* extra copies. This mutation decreased amount of Pho87 in both species (Fig. 3d,e), while one extra copy of *ABV1* could even slightly increase it (Fig. 3f; Table 1), despite similar effects of these modifications on vanadate resistance. This demonstrates that, in addition to the Pho87-independent effects, Abv1 influences phosphate homeostasis *via* affecting Pho87 level.

Summarizing the data presented in this section, it can be noted that *ABV1* expression affects regulation of *PHO84* and *PHO87*. The *ABV1* effect on the phosphate homeostasis was also revealed by observing acid phosphatase derepression in a strain with increased *ABV1* dosage.

## Discussion

In this work, we have identified and characterized three genes which influence vanadate resistance in *O*. *polymorpha* and *O*. *parapolymorpha*. One of these genes, designated as its *S*. *cerevisiae* orthologue, *PHO87*, encodes a protein with a dual function, namely the low affinity plasma membrane P_i_ transporter and sensor of external P_i_ concentration. Involvement of Pho87 in modulation of vanadate resistance indicates that vanadate, as a P_i_ analog, enters the cell *via* the P_i_ transport system. Being a P_i_ transporter, Pho87 mediates vanadate uptake, while, being an external P_i_ sensor, it can provide protection against vanadate by repressing high affinity phosphate transporters. Indeed, inactivation of *PHO87* led to a substantial increase of vanadate resistance at low P_i_ concentrations, but narrowed down the effect of external P_i_ in rescuing cells from vanadate toxicity. This correlates with the effect of *PHO87* inactivation on regulation of the *PHO84* gene coding for a high affinity phosphate transporter.

Incubation in P_i_-depleted medium leads to a drastic decrease in the amount of Pho87 (the full-length protein becomes undetectable by immunoblotting), most probably due to activation of its degradation, as observed in *S*. *cerevisiae*^7^. Nevertheless, some amount of functional Pho87 should be retained in the cell, since it essentially influences vanadate resistance and *PHO84* regulation. Remarkably, higher Pho87 level at high P_i_ concentration does not provide efficient vanadate uptake indicating that its transporter function is strongly inhibited.

Another identified gene is a homologue of *S*. *cerevisiae MNN4* designated here as *ABV1*. Like its *S*. *cerevisiae* homolog, *ABV1* is responsible for mannosylphosphate attachment to glycoside chains of proteins passing through the secretory pathway, since its inactivation abolishes Alcian blue binding to the cell surface. Although it was identified as a gene whose inactivation decreases vanadate resistance in the vanadate resistant species *O*. *polymorpha*, this mutation has the opposite effect in *O*. *parapolymorpha* (the vanadate sensitive species). The increased vanadate resistance in the *O*. *parapolymorpha abv1-Δ* mutant can be mediated by the decrease in Pho87 level, since Pho87 confers pronounced vanadate sensitivity in this yeast. Indeed, the effect of the *abv1-Δ* mutation on vanadate resistance in the *O*. *parapolymorpha* strain lacking *PHO87* is almost indistinguishable. At the same time, *ABV1* extra copies are able to increase vanadate resistance in *O*. *parapolymorpha* independently of the presence of *PHO87*. This effect in the *pho87-Δ* strain can be easily explained, since it correlates with much lower activity of the promoter of the *PHO84* gene coding for the high affinity plasma membrane P_i_ transporter. However, in the strain bearing wild-type *PHO87*, the increase in vanadate resistance in response to *ABV1* extra copies seems to be puzzling, since they do not lead to significant repression of the *PHO84* promoter, but increase Pho87 level. This suggests that the Pho87 transporter function is essentially inhibited since otherwise vanadate resistance would be decreased.

*O*. *polymorpha* Pho87 seems to be less effective in conferring vanadate uptake compared to *O*. *parapolymorpha* Pho87. This, along with higher activity of Abv1, can determine the difference in vanadate resistance between these two yeast species. Indeed, *O*. *polymorpha* strain lacking *ABV1* and expressing *OpaPHO87* becomes almost as sensitive to vanadate as *O*. *parapolymorpha* wild type strain and *vice versa*, *O*. *parapolymorpha* expressing *OpoPHO87* instead of its own gene and bearing *OpoABV1* is as resistant as *O*. *polymorpha* (Supplementary Fig. S3). One can assume that high vanadate resistance is achieved when Pho87 is efficient as a sensor but not as a transporter, providing efficient repression of genes for the high affinity transporters, while high activity of Abv1 supports this repression *via* a Pho87 independent pathway. Inability of *ABV1* inactivation to reduce vanadate resistance in *O*. *parapolymorpha pho87-Δ* strain could be due to its naturally lower activity, which is insufficient to affect this Pho87 independent regulatory pathway. However, the present study does not provide experimental data which could solidly prove this suggestion.

The third identified gene affecting vanadate resistance codes for the Ypt6 GTPase involved in vesicle transport between secretory organelles including endosomes. This gene is unlikely to determine the difference between *O*. *polymorpha* and *O*. *parapolymorpha*, since its inactivation noticeably affects viability of both species. One could suggest that it influences vanadate resistance *via* glycosylation since its inactivation decreases Alcian blue staining and affects vanadate resistance in different genetic backgrounds similar to the *abv1-Δ* mutation. However, its effect on the *PHO84* promoter was opposite to the effect of *ABV1*, which contradicts this suggestion. Theoretically, the loss of Ypt6 may influence endocytosis of the plasma membrane phosphate transporters by affecting endosome dynamics.

Summarizing the obtained data, we can conclude the following: vanadate is absorbed *via* the P_i_ transport system. Pho87 has a dual role in resistance to vanadate: it is involved in vanadate uptake as a P_i_ transporter and can provide protection against vanadate by repressing genes for other P_i_ transporters as an external P_i_ sensor. The role of mannosylphosphate attachment to protein glycoside chains is also complex. On one hand, it increases Pho87 level, which in turn has a dual effect on vanadate resistance, since Pho87 confers vanadate uptake as a transporter and represses *PHO84* expression as a P_i_ sensor. On the other hand, increased expression of *ABV1* deepens repression of *PHO84 via* a Pho87-independent pathway.

## Methods

### Genetic nomenclature and genetic manipulation techniques

Standard genetic nomenclature was used. For convenience, where necessary, *O*. *polymorpha* and *O*. *parapolymorpha* gene names were precluded with “*Opo*” or “*Opa*”, respectively.

Mutagenesis by the random integration of linear DNA fragments^35^ and following identification of mutant loci were performed in *O*. *polymorpha* strains u23M25 and m14 strains as described previously^36^. Vanadate sensitive mutants were selected by replica plating of transformants onto YPD plates supplemented with 3 mM Na_3_VO_4_. *O*. *parapolymorpha* DL1-L strain^37^ was mutagenized by random integration of the PvuII-linearized pKAM554A plasmid^38^. After transformation cells were overnight incubated in liquid YPD medium containing G418 (100 mg/L) and then were spread onto YPD plates supplemented with 5 mM Na_3_VO_4_. Obtained colonies were confirmed for G418 resistance. One of obtained clones demonstrated stable increase in the vanadate resistance and was resistant to G418. Its DNA was digested with HincII (no sites in the vector) and NruI (unique site in the vector) and treated with T4 DNA ligase. The ligation mix was used as a template for PCR with primers flanking the NruI site in the vector to amplify the fragment possessing portion of the integration locus, which was then sequenced and compared with *O*. *parapolymorpha* genome database by the BLAST search.

### Yeast strains and plasmid construction

The *O*. *parapolymorpha* DLdaduA strain (*leu2 ade2-Δ ura3::ADE2*) was obtained by sequential inactivation of *ADE2* and then *URA3* in the DL1-L strain^37^. Manifestations of alterations in the *PHO87*, *ABV1*, and *YPT6* genes were studied in transformants of the DLdaduA strain, its derivatives DLdaduA-R and DLdaduA-Z bearing *Renilla* luciferase gene under control of *OpaPHO84* promoter (*P*_*PHO84*_*-Rluc*) or *E*. *coli lacZ* gene under control of *OpaPHO87* promoter (*P*_*PHO87*_*-lacZ*), respectively, and *O*. *polymorpha* 1B27 (*leu2 ade2 ura3::ADE2*) strain^39^. Genotypes of these transformants are presented in the Supplementary Table S1.

The integrative vector pCCUR1 was constructed by insertion of BamHI-BamHI fragment of the *OpoURA3* locus into the BamHI site of the pBCKS(+) vector (Agilent, USA). This vector was then modified by deletion of BglII-XhoI fragment followed by removing EcoRI site using the Klenow enzyme to obtain pCAI24 plasmid. The *OpoPHO87* and *OpaPHO87* genes were obtained by PCR using primers 5′-CAGGC TGGAT GTGAT GTTG-3′ and 5′-GCTGC GACAT CTTCA TCAC-3′. The *OpaPHO87* PCR product was inserted into the SmaI site of pKAM556^38^ to obtain the pKAM585 plasmid. The latter was modified by insertion of *OpoURA3* between Asp718 and PstI sites outside *PHO87* to obtain pKAM591 plasmid. To obtain the *OpaPHO87* disruption cassette, BamHI-BamHI 3367 bp fragment of pKAM585 was self-ligated, digested with BsrGI and StuI, and inserted between BsrGI and PsiI sites of pCAI24. Linearization of resulting pCAM599 plasmid with BamHI created the disruption cassette whose integration into the genome *via* double crossover recombination led to replacement of the BsrGI-StuI fragment of *PHO87* ORF for the pCAI24 vector sequence.

The DLdaduA-R and DLdaduA-Z strains possessed pKAZ15 or pKAM615 plasmids, respectively, which were integrated into unidentified loci. The pKAZ15 plasmid was obtained by sequential insertion into the pKAM554A vector polylinker the sequence coding for the *Renilla* luciferase recovered from the pDB688 plasmid^40^ and the sequence of the *OpaPHO84* promoter region obtained by PCR using primers 5′-TTGAA TTCGG CCATA AGAAA GATG-3′ and 5′-TCGGA TCCTC TGGCT CACTC-3′. The pKAM615 plasmid was obtained by replacement of HindIII-EheI fragment of pKAM585 for HindIII-PsiI fragment of Yep356R bearing the *E*. *coli lacZ* coding sequence^41^. The DLdaduA strain was transformed either with Psp5II-linearized pKAZ15 or with Bsp119I-linearized pKAM615 to obtain integrative G418-resistant transformants, which were confirmed for expression of *Renilla* luciferase or β-galactosidase, respectively.

The *ABV1* gene was inactivated using plasmid, which was recovered from the *O*. *polymorpha abv1* mutant obtained by random integration of the pCAD24 plasmid possessing *OpoLEU2* as a selectable marker^36^. To do this, chromosomal DNA of this mutant was digested with BsrGI, which does not cut within the pCAD24 sequence, and self-ligated. The plasmid obtained from this ligateion by *E*. *coli* transformation was designated as pCAM468. It possessed *O*. *polymorpha LEU2* gene as a selectable marker and fragments of the *ABV1* gene conferring integration of the BsrGI-digested plasmid into the corresponding chromosomal locus *via* double crossing over homologous recombination. If transformation with a *LEU2*-carrying plasmid was required after *ABV1* inactivation, the disruption cassettes based on a vector, which can be self-excised by the Cre-*loxP*-mediated recombination^42,43^, was used. This cassette was constructed by insertion of the SalI-BalI 1.1 kb pCAM468 fragment between the SalI and EcoRV sites of the pAM619 vector^42^.

To obtain strains possessing extra copies of *ABV1* three different plasmids were used. The pAM467 plasmid was isolated from a *O*. *polymorpha* genomic library based on the AMIpSL1 vector^44^ by complementation of vanadate sensitivity of the *abv1* mutant obtained by random integration. The pKAB1 plasmid was constructed by insertion of PciI-MluI fragment of pAM467 between NcoI-MluI sites of the pBGX1 plasmid consisting of the pUK21 *E*. *coli* vector^45^ and XhoII-XhoII fragment of the *O*. *polymorpha LEU2* locus cloned into the BglII site. The *O*. *parapolymorpha ABV1* was obtained by PCR with primers 5′-AAACG CGTAC TCTGA CAGCG AC-3′ and 5′-ATTCT GGGCG GGTTC ACG-3′ and cloned between NdeI and MluI sites of the AMIpSL1 vector to obtain the pAZ16 plasmid. The AMIpSL1-based plasmids were used for yeast transformation in circular form. This led to obtaining transformants bearing autonomously replicating plasmids. Most rapidly growing sub-clones of such transformants, obtained after several round of selection, as a rule, possess the plasmid integrated into the genome in several copies^44^. The pKAB1 plasmid was linearized by PstI within the selectable marker *O*. *polymorpha LEU2* to target the plasmid integration into this locus in a single copy.

To obtain prototrophic transformants, strains were transformed with empty vectors pCHLX^37^, pCCUR1 and/or AMIpSL1, when required.

### Culture media

Apart from the regular yeast media YPD (1% yeast extract, 2% peptone, 2% glucose) and SC-D (0,67% Yeast Nitrogen Base, 2% glucose), complex medium depleted of phosphate was also used. The latter was prepared according previously reported approach^46^ with slight modifications as follows: 5 g of yeast extract and 10 g of peptone were dissolved in 170 ml of distilled water by autoclaving. Then 2.46 g of MgSO4 was dissolved in the obtained solution. The solution was supplemented with 7 ml of 5 M NaOH to obtain precipitate, which was removed first by centrifugation and then by filtration through a 0.2 µM filter. pH was adjusted to 7.2 by concentrated HCl, then water was added to a final volume of 200 ml to obtain 5X concentrate of the medium without carbon source. This concentrate was autoclaved, filtered again and used to prepare 1X media supplemented with 2% glucose and 2% agar if required. When necessary, this medium was supplemented with Na-P_i_ buffer pH7.2 to achieve a required phosphate concentration. Phosphate concentration in YPD calculated from the BD Biosciences technical data on phosphate content in yeast extract and peptone (https://www.bd.com/documents/guides/user-guides/DS_CM_Bionutrients-technical-manual_UG_EN.pdf) was approximately 4 mM. Vanadate was added to complex media as filter-sterilized 100 mM Na_3_VO_4_ water solution.

### Immunoblotting

Prior to immunoblotting proteins were resolved by the SDS-electrophoresis in 10% acrylamide gels^47^. To analyze intracellular carboxypeptidase Y, samples were prepared using the alkaline extraction method^48^ from equal amount of cells, which was determined by assaying total cellular protein^49^. Previously described antiserum^50^ was used to detect CPY. Samples for Pho87 analysis were obtained using a special enrichment procedure^51^. Antibody from antisera obtained from rabbits immunized with the *E*. *coli*-expressed *O*. *parapolymorpha* Pho87 fragment were affinity purified by binding to the protein used for immunization. Prior the performing immunoblotting, samples were equalized according to density of major bands on Coomassie Blue-stained gels. The regular (New England BioLabs (NEB), cat. # P7702) and pre-stained protein markers (ThermoFisher Scientific, cat. #sm0441) were used in Coomassie Blue-stained gels and immunoblots, respectively.

### Enzyme activity and ^32^P_i_ uptake assays

β-Galactosidase and acid phosphatase activities were assayed using chromogenic substrates o-nitrophenyl-β-D-galactopyranoside^52^ and p-nitrophenylphosphate^53^, respectively. *Renilla* luciferase was assayed using commercial kit (Biotium Inc., cat. #30004-1). The values were normalized to the total cellular protein.

The ^32^P_i_ uptake was assayed according to the previously described protocol^33^ as follows. Cells cultures grown overnight in YPD were 20-fold diluted with the same medium and grown additionally for 4 h. Cells from 1 ml of obtained cultures were harvested by centrifugation, washed with 3% glucose solution containing 25 mM Tris-succinate buffer pH4.5 or 25 mM Tris-succinate buffer pH8.0 and 10 mM NaCl, and re-suspended in 30 µL of the same buffer. The uptake was initiated by adding 1 µL of [^32^P] orthophosphate (2.4 Ci L^−1^, 50 Ci mmol^−1^) to the cell suspension. Phosphate uptake was terminated by addition of 1 mL of ice-cold Tris-succinate dilution buffer. The cell suspensions were immediately filtered through the Whatman GF/F filters (Whatman, UK) and washed with the same cold dilution buffers. The radioactivity retained on the filters was determined by liquid scintillation spectrometry.

### Sources of reagents and media components

Reagents and media components were purchased from following suppliers: Difco (Yeast Nitrogen Base, Peptone, Yeast Extract), Sigma (various salts and buffer components). Enzymes and kits for *in vitro* DNA manipulations were supplied by NEB, Thermo Scientific, and Sibenzyme.

## Electronic supplementary material

Supplementary figures and table

## Data Availability

All data generated or analyzed during this study are included in this published article and its Supplementary Information.
