# Supplementary material for: Dissection of differential vanadate sensitivity in two Ogataea species links protein glycosylation and phosphate transport regulation
Source: Sci Rep. 2018 Nov 6;8:16428. doi: 10.1038/s41598-018-34888-5 (PMC6219546; doi:10.1038/s41598-018-34888-5)
Supplement: Supplementary file 1 — Supplementary figures and table [file 41598_2018_34888_MOESM1_ESM.pdf]

Dissection of differential vanadate sensitivity in two *Ogataea* species links protein glycosylation and phosphate transport regulation.

Azamat V. Karginov, Anastasia V. Fokina, Hyun Ah Kang, Tatyana S. Kalebina, Tatyana A. Sabirzyanova, Michael D. Ter-Avanesyan, Michael O. Agaphonov*

*** Corresponding author:** Michael Agaphonov, Bach Institute of Biochemistry, Research Center of Biotechnology RAS, Leninsky ave., 33, build. 2, 119071 Moscow, Russian Federation. Phone: +7 (495) 9545283; fax: +7 (495) 9542732 E-mail: agaphonov@inbi.ras.ru

Supplementary materials

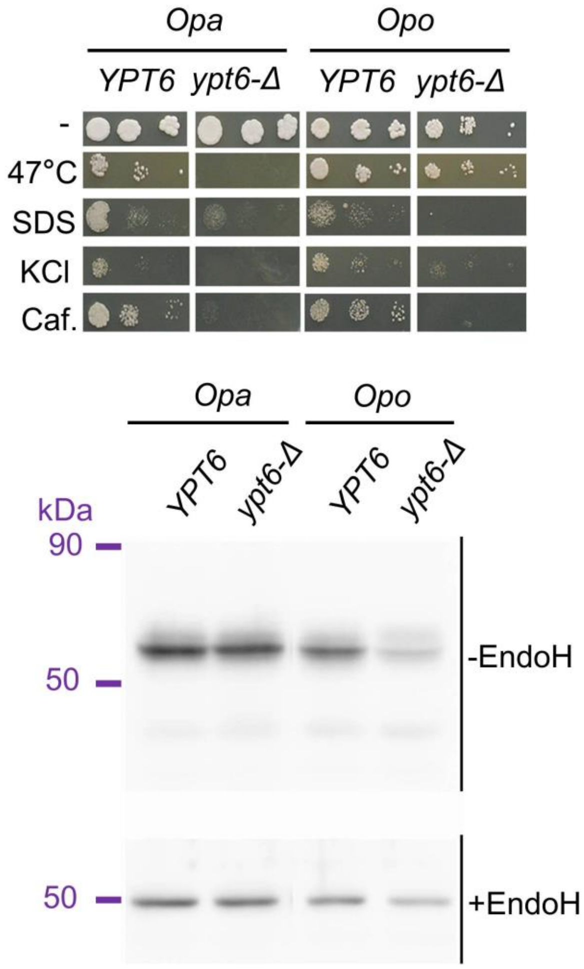

Supplementary Figure S1. Effects of inactivation of *YPT6* in *O.polymorpha (Opo*) and *O. parapolymorpha (Opa*). Upper panel, growth under different stress conditions. 10-fold serially diluted cell suspensions were applied onto YPD medium supplemented with 0,008% SDS (SDS), 0.5M KCl (KCl), or 10 mM caffein (Caf.). Control plate (-) and the plate incubated at 47°C contained YPD without additives. Lower panel, immunoblotting of intracellular CPY. +EndoH and -EndoH, samples treated or untreated with endoglycosydase H, respectively. Numbers of transformants in the Supplementary Table S1 are following: *O. polymorpha YPT6* and *ypt6-Δ*, 58 and 62, respectively; *O. parapolymorpha YPT6* and *ypt6-Δ*, 6 and 8, respectively.

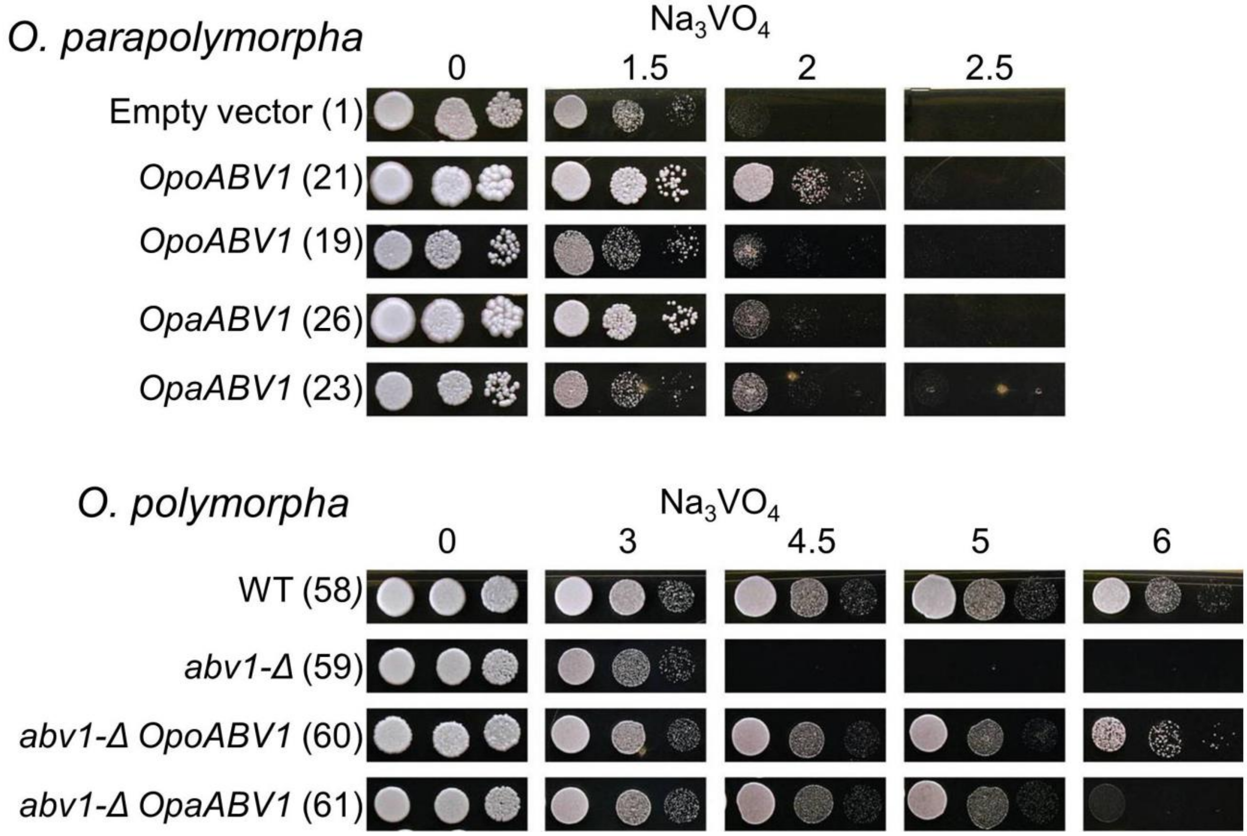

Supplementary Figure S2. Effects of plasmids bearing *OpoABV1* or *OpaABV1* on sensitivity to vanadate in *O. polymorpha abv1-Δ* mutant and *O. parapolymorpha*. Cell suspensions were 10-fold serially diluted and spotted onto YPD medium supplemented with different concentrations of Na_3VO_4. Numbers in brackets correspond to designation of transformants in the Supplementary Table S1.

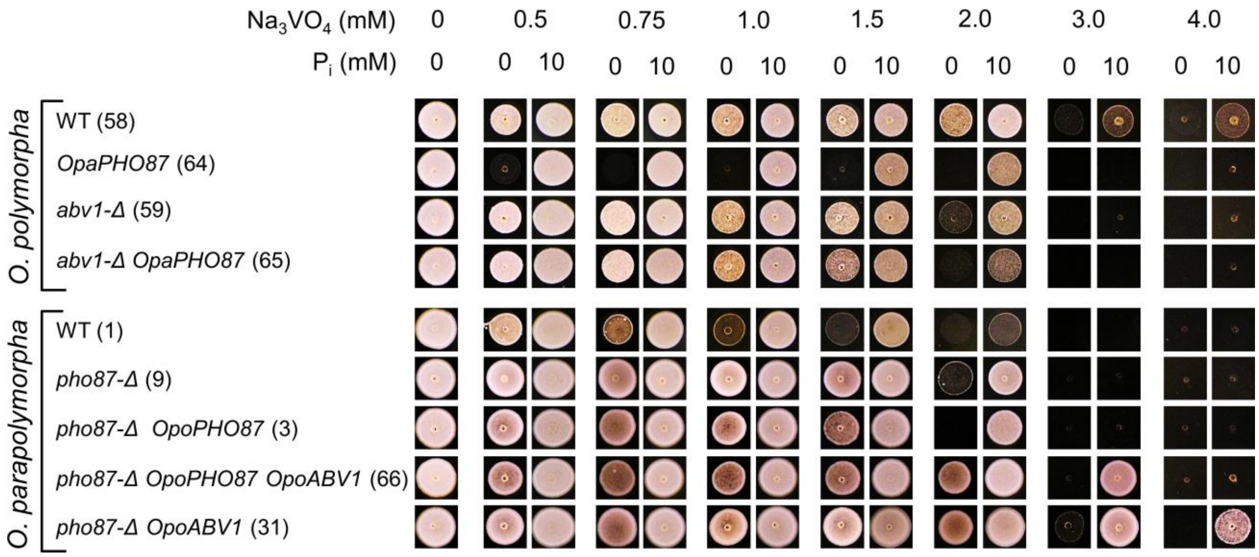

Supplementary Figure S3. Dependence of vanadate sensitivities of *O. polymorpha* and *O. parapolymorpha* transformants expressing the heterologous *PHO87* genes on *ABV1*. Numbers in brackets correspond to designation of transformants in the Supplementary Table S1. Overnight saturated cultures grown in the phosphate depleted medium supplemented with 0.5 mM Na-P_i buffer were diluted 250-fold and spotted onto plates with phosphate depleted medium supplemented with different concentrations of Na_3VO_4 and Na-P_i buffer (pH 7.2). Plates were incubated at 37°C for two days.

Supplementary Table S1. Yeast transformants. Round and square brackets in genotypes mark genes of an integrated or autonomously replicating plasmid, respectively. Plasmid names are indicated in superscript.

#	parent strain	Genotype and comments
		O. parapolymorpha
1	DLdaduA	*leu2 ade2-Δ ura3::ADE2* (*URA3*)^{pCCUR1} (*LEU2*)^{pCHLX}
2, 3	DLdaduA	*leu2 ade2-Δ ura3::ADE2 pho87::URA3* (*LEU2*)^{pCHLX} (*G418*^{R} *OpoPHO87*)^{pKAZ1A}
4, 5	DLdaduA	*leu2 ade2-Δ ura3::ADE2 pho87::LEU2* (*URA3 OpaPHO87*)^{pKAM591}
6	DLdaduA-R	*leu2 ade2-Δ ura3::ADE2* (P_{PHO84}*-Rluc G418*^{R})^{pKAZ15} (*URA3*)^{pCCUR1} (*LEU2*)^{pCHLX}
7	DLdaduA-R	*leu2 ade2-Δ ura3::ADE2 abv1::LEU2* (P_{PHO84}*-Rluc G418*^{R})^{pKAZ15} (*URA3*)^{pCCUR1}
8	DLdaduA-R	*leu2 ade2-Δ ura3::ADE2 ypt6::LEU2* (P_{PHO84}*-Rluc G418*^{R})^{pKAZ15} *(URA3)*^{pCCUR1}
9	DLdaduA-R	*leu2 ade2-Δ ura3::ADE2 pho87::URA3 (*P_{PHO84}*-Rluc G418*^{R}*)*^{pKAZ15} (*LEU2*)^{pCHLX}
10-13	DLdaduA-R	*leu2 ade2-Δ ura3::ADE2* (P_{PHO84}*-Rluc G418*^{R})^{pKAZ15} (*LEU2*)^{pCHLX} (*OpaPHO87 URA3*)^{pKAM591} Obtained by integration of BglII-digested pKAM591 into unidentified genomic loci.
14-16	DLdaduA-R	*leu2 ade2-Δ ura3::ADE2* (P_{PHO84}*-Rluc G418*^{R})^{pKAZ15}

		(*URA3*)^{pCCUR1} (*OpoABV1 LEU2*)^{pKAB1}<br>Obtained by integration of PstI-digested pKAB1 presumably into the *LEU2* locus in a single copy.
17-20	DLdaduA-R	*leu2 ade2-Δ ura3::ADE2* (*P*$_{PHO84}$*-Rluc G418*^{R})^{pKAZ15} (*URA3*)^{pCCUR1} (*ScLEU2 OpoABV1*)^{pAM467}<br>Obtained from transformants bearing autonomous pAM467 by selection of subclones, in which the plasmid was integrated presumably into a telomere locus in several copies.
21	DLdaduA-R	*leu2 ade2-Δ ura3::ADE2* (*P*$_{PHO84}$*-Rluc G418*^{R})^{pKAZ15} (*URA3*)^{pCCUR1} [*ScLEU2 OpoABV1*]^{pAM467}<br>A transformant bearing autonomously replicating pAM467
22-25	DLdaduA-R	*leu2 ade2-Δ ura3::ADE2* (*P*$_{PHO84}$*-Rluc G418*^{R})^{pKAZ15} (*URA3*)^{pCCUR1} (*ScLEU2 OpaABV1*)^{pAZ16}<br>Obtained from transformants bearing autonomous pAZ16 by selection of subclones, in which the plasmid was integrated presumably into a telomere locus in several copies.
26	DLdaduA-R	*leu2 ade2-Δ ura3::ADE2* (*P*$_{PHO84}$*-Rluc G418*^{R})^{pKAZ15} (*URA3*)^{pCCUR1} [*ScLEU2 OpaABV1*]^{pAZ16}<br>A transformant bearing autonomously replicating pAZ16
27	DLdaduA-R	*leu2 ade2-Δ ura3::ADE2 abv1::loxP ypt6::LEU2* (*P*$_{PHO84}$*-Rluc G418*^{R})^{pKAZ15} (*URA3*)^{pCCUR1}
28	DLdaduA-R	*leu2 ade2-Δ ura3::ADE2 pho87::URA3 abv1::LEU2* (*P*$_{PHO84}$*-Rluc G418*^{R})^{pKAZ15}
29	DLdaduA-R	*leu2 ade2-Δ ura3::ADE2 pho87::URA3 ypt6::LEU2* (*P*$_{PHO84}$*-Rluc G418*^{R})^{pKAZ15}

30	DLdaduA-R	*leu2 ade2-Δ ura3::ADE2 pho87::URA3 abv1::loxP ypt6::LEU2* (P_{PHO84}-*Rluc G418*^{R})^{pKAZ15}
31-36	DLdaduA-R	*leu2 ade2-Δ ura3::ADE2 pho87::URA3* (P_{PHO84}-*Rluc G418*^{R})^{pKAZ15} (*OpoABV1 LEU2*)^{pKAB1}<br>Obtained by integration of PstI-digested pKAB1 presumably into the *LEU2* locus in a single copy.
37-40	DLdaduA-R	*leu2 ade2-Δ ura3::ADE2* (P_{PHO84}-*Rluc G418*^{R})^{pKAZ15} *pho87::URA3* (*ScLEU2 OpoABV1*)^{pAM467}<br>Obtained from transformants bearing autonomous pAM467 by selection of subclones, in which the plasmid was integrated presumably into a telomere locus in several copies.
41-44	DLdaduA-R	*leu2 ade2-Δ ura3::ADE2 pho87::URA3* (P_{PHO84}-*Rluc G418*^{R})^{pKAZ15} (*OpaABV1 ScLEU2*)^{pAZ16}<br>Obtained from transformants bearing autonomous pAZ16 by selection of subclones, in which the plasmid was integrated presumably into a telomere locus in several copies.
45	DLdaduA-Z	*leu2 ade2-Δ ura3::ADE2* (P_{PHO87}-*lacZ G418*^{R})^{pKAM615} (*URA3*)^{pCCUR1} (*LEU2*)^{pCHLX}
46	DLdaduA-Z	*leu2 ade2-Δ ura3::ADE2 abv1::LEU2* (P_{PHO87}-*lacZ G418*^{R})^{pKAM615} (*URA3*)^{pCCUR1}
47	DLdaduA-Z	*leu2 ade2-Δ ura3::ADE2* (P_{PHO87}-*lacZ G418*^{R}) ^{pKAM615} *pho87::URA3* (*LEU2*)^{pCHLX}
48-50	DLdaduA-Z	*leu2 ade2-Δ ura3::ADE2* (P_{PHO87}-*lacZ G418*^{R})^{pKAM615} (*URA3*)^{pCCUR1} (*OpoABV1 LEU2*)^{pKAB1}<br>Presumably, single copy integration of pKAB1 into the *LEU2*

		locus
51-54	DLdaduA-Z	*leu2 ade2-Δ ura3::ADE2* (*P_{PHO87}-lacZ G418^R*)^{pKAM615} (*URA3*)^{pCCUR1} (*ScLEU2 OpoABV1*)^{pAM467}
55-57	DLdaduA-Z	*leu2 ade2-Δ ura3::ADE2* (*P_{PHO87}-lacZ G418^R*)^{pKAM615} (*LEU2*)^{pCHLX} (*URA3 OpaPHO87*)^{pKAM591}
66	DLdaduA	*leu2 ade2-Δ ura3::ADE2 pho87::URA3* (*G418^R OpoPHO87*)^{pKAZ1A} (*OpoABV1 LEU2*)^{pKAB1}
		O. polymorpha
58	1B27	*leu2 ade2 ura3::ADE2* (*URA3*)^{pCCUR1} (*LEU2*)^{pCHLX}
59	1B27	*leu2 ade2 ura3::ADE2 abv1::loxP* (*URA3*)^{pCCUR1} (*LEU2*)^{pCHLX}
60	1B27	*leu2 ade2 ura3::ADE2 abv1::loxP* (*URA3*)^{pCCUR1} (*OpaABV1 ScLEU2*)^{pAZ16}
61	1B27	*leu2 ade2 ura3::ADE2 abv1::loxP* (*URA3*)^{pCCUR1} (*ScLEU2 OpoABV1*)^{pAM467}
62	1B27	*leu2 ade2 ura3::ADE2 ypt6::LEU2* (*URA3*)^{pCCUR1}
63	1B27	*leu2 ade2 mox::uPA ura3::ADE2* (*URA3*)^{pCCUR1} (*LEU2*)^{pCHLX} (*G418^R OpoPHO87*)^{pKAZ1A}
64	1B27	*leu2 ade2 mox::uPA ura3::ADE2* (*LEU2*)^{pCHLX} (*URA3 OpaPHO87*)^{pKAM591}
65	1B27	*leu2 ade2 ura3::ADE2 abv1::loxP* (*LEU2*)^{pCHLX} (*URA3 OpaPHO87*)^{pKAM591}

Supplementary Table S2. [^{32}P]Orthophosphate uptake activity (10^{-4} mmol min^{-1} g^{-1} cells dry weight). Numbers in brackets correspond to designation of transformants in the Supplementary Table S1.

Strain	pH4.5			pH8.0 10mM NaCl		
	0’	1’	5’	0’	1’	5’
WT (1)	0.65±0.26	6,4±0.6	35±9	0.61±0,09	3.3±0.5	7.0±1.7
pho87-Δ (9)	0.53±0.12	4.5±1.0	20±6	0.87±0.20	4.5±1.3	9.4±3.0
